# Effect of Laser Energy Density on Surface Morphology, Composition and Cleaning Mechanism of TC1 Titanium Alloy During Nanosecond Laser Cleaning

**DOI:** 10.3390/ma19091695

**Published:** 2026-04-22

**Authors:** Yang Chen, Haixiang Sun, Xuecheng Li, Hongyan Song, Zexuan Han, Jinhao Nie, Donghe Zhang, Jie Xu, Bin Guo

**Affiliations:** 1 Key Laboratory of Micro-Systems and Micro-Structures Manufacturing of Ministry of Education, Harbin Institute of Technology, Harbin 150080, China; 24s109311@stu.hit.edu.cn (Y.C.); sunhaixiang0075@163.com (H.S.); 24b909033@stu.hit.edu.cn (H.S.); 25b909121@stu.hit.edu.cn (Z.H.); 15230570237@163.com (J.N.);; 2 School of Materials Science and Engineering, Harbin Institute of Technology, Harbin 150080, China; 3 AECC Harbin Dong’an Engine Co., Ltd., Harbin 150060, China; lxchit@163.com; 4 Zhengzhou Advanced Research Institute of Harbin Institute of Technology, Zhengzhou 450000, China

**Keywords:** titanium alloy, laser cleaning, oxide layer, mechanism

## Abstract

**Highlights:**

**What are the main findings?**

- After cleaning at a pressure of 6.37 J/cm^2^, the oxygen content on the surface decreased to 4.87%.
- The surface roughness was as low as 0.37 µm, and the microhardness was 268.9 HV.
- The mechanism of oxide layer laser removal mainly involves laser ablation induced by plasma impact.

**What are the implications of the main findings?**

- Revealed the optimal process parameters.
- Reduces the roughness while having a minimal impact on hardness.
- The mechanism of oxide layer removal is explained.

**Abstract:**

To remove the oxide layer of TC1 titanium alloys in an environmentally friendly and efficient manner, this study conducted experiments using a nanosecond pulsed laser to systematically investigate the influence of different laser energy densities on the cleaning effect. The results showed that the oxide layer could be completely removed at an energy density of 6.37 J/cm^2^, with the surface oxygen element content reduced to 4.87%. The macroscopic surface presented a silvery metallic luster. Moreover, the roughness decreased significantly with the increase in energy density. At 6.37 J/cm^2^, the surface roughness dropped to 0.37 µm. The mechanism of removing the oxide layer of TC1 titanium alloy mainly includes laser ablation and plasma impact. At energy densities ranging from 2.55 J/cm^2^ to 6.37 J/cm^2^, the cleaning mechanism was mainly laser ablation. When the energy density exceeded 6.37 J/cm^2^, the cleaning mechanism gradually shifted from laser ablation to plasma impact as the dominant factor. Meanwhile, the microhardness of the samples after laser cleaning was basically consistent with that of the samples subjected to mechanical grinding, which provides a basis for a nanosecond pulsed laser to replace traditional methods for oxide layer cleaning.

## 1. Introduction

Titanium alloys, as metals characterized by high specific strength, excellent corrosion resistance, and favorable high-temperature performance [1], are widely utilized in aerospace, biomedical, and marine engineering fields [2,3,4]. TC1 (Ti–2Al–1.5Mn), as a representative near-α titanium alloy, has garnered increasing attention from both industry and academia due to its outstanding comprehensive properties. Its welding performance is particularly remarkable, featuring well-formed welds, a narrow heat-affected zone, and low cold cracking sensitivity. Under optimized welding parameters, the joint strength can approach that of the base metal, making it an ideal material for integrated welding manufacturing of complex components. However, weld quality is highly sensitive to the pre-weld surface condition. Trace surface contaminants such as oxide layers, grease, and moisture readily induce porosity, inclusions, and brittle phase precipitation, significantly reducing the service reliability of welded joints [5,6]. Therefore, pre-weld surface cleaning is a core preliminary process for ensuring high-quality welding. Traditional pre-welding cleaning methods primarily rely on manual grinding and chemical cleaning. Manual grinding [7] not only involves lengthy cleaning cycles and high labor intensity but also struggles to maintain consistency and risks damaging the substrate. Chemical cleaning [8,9], meanwhile, poses environmental pollution and health hazards to workers. Consequently, there is an urgent need for an eco-friendly and efficient cleaning approach. Laser cleaning, as a high-precision, pollution-free method, offers a novel solution for pre-welding surface cleaning of titanium alloys.

Laser cleaning is hailed as one of the most promising eco-friendly cleaning technologies of the 21st century [10] and has been widely applied to remove oxide layers [11,12], paint [13,14], coatings [15,16,17], marine microorganisms [18], and more. Laser cleaning is a non-contact precision processing technology that utilizes high-energy-density laser beams to irradiate contaminants on a substrate surface, inducing physicochemical reactions such as melting, vaporization, and stripping to achieve surface cleaning. Compared to manual polishing, laser cleaning involves no physical contact and features adjustable parameters, ensuring uniformity and consistency in surface treatment. Compared to chemical cleaning, laser cleaning better aligns with the requirements of green precision manufacturing, offering advantages such as no consumables, high efficiency, and ease of automation integration. In the aerospace field, the removal of the oxide layer on the surfaces of titanium alloy and high-temperature alloy components has always been a challenge. Laser removal of the oxide layer can achieve the complete stripping of the oxide layer while ensuring the intact surface morphology of the substrate, providing a new technical path for green and high-precision surface treatment of critical aerospace components. In the biomedical field, the surface cleanliness and smoothness of medical implants (such as titanium alloy artificial joints) directly determine their biocompatibility. Laser cleaning, as a physical cleaning method without chemical reagents, can ensure the absolute cleanliness of the implant surface and avoid secondary pollution, and is expected to be widely applied in the surface activation treatment of precision medical devices. In recent years, numerous scholars have conducted research on laser cleaning of oxide layers. Zhao et al. [19] employed a nanosecond pulsed laser to clean oxide scale from the surface of SUS301L stainless steel joints. Using a two-step method—first cleaning with a Gaussian beam followed by a top-hat beam—they effectively removed the oxide scale, revealing a smooth, silvery-white surface while enhancing the joint’s surface corrosion resistance. Guo et al. [20] employed a pulsed laser to clean the oxide layer on the surface of 6061 aluminum alloy. Combining experimental and simulation results, they found that the evolution of surface morphology during laser cleaning of aluminum alloys is primarily related to back pressure, surface tension, and gravity. At an average power of 20 W, the maximum flow velocity reached only 5 m/s, whereas at average powers of 40 W and 60 W, the maximum flow velocity attained 150 m/s. Li et al. [21] employed a nanosecond pulsed laser to clean the oxide layer on the surface of TA15 titanium alloy. Through a combination of simulation and experimentation, they found that at a maximum power density of 31.85 MW/cm^2^ and a spot overlap ratio of 80%, the surface quality remained relatively smooth and crack-free. After cleaning, a dense, uniform oxide layer approximately 29 nm thick formed on the titanium alloy surface. The primary removal mechanism at this point was laser ablation. Wang et al. [22] investigated the effects of laser energy density on the surface morphology, microhardness, and corrosion resistance of 7075 aluminum alloy. The study revealed that when the energy density ranged from 1.43 J/cm^2^ to 1.82 J/cm^2^, it could enhance the impact resistance of the cleaned surface while maximally preserving the alloy’s corrosion resistance, resulting in a 5.62% increase in surface microhardness. Sun et al. [23] used a nanosecond pulsed laser to remove the oxide film on the surface of TC1 titanium alloy. They systematically evaluated the surface conditions of the substrates after different energy cleaning processes. The study found that the laser cleaning samples achieved the best removal effect at 3.76 J/cm^2^, and exhibited better mechanical properties, with a 15.2% increase in microhardness and a 71.3% decrease in wear rate.

In conclusion, although many scholars have conducted extensive research on laser removal of metal oxide films, including studies on oxide films of titanium alloys such as TC1 and TA15, the different alloy compositions in TC1 result in different physical properties (melting point, thermal conductivity), and TC1 is mainly used in welding structures and is more sensitive to the residual oxygen content and morphology on the surface. Therefore, conducting research on laser removal of oxide films for TC1 titanium alloys is of great significance.

This study employs a nanosecond pulsed laser to clean the oxide layer of TC1 titanium alloy. The effects of different laser energy densities on the macroscopic morphology were investigated. Cleaning efficacy was evaluated by combining SEM microstructure analysis and EDS energy spectrum data at various laser energy densities. X-ray photoelectron spectroscopy (XPS) was employed to investigate chemical state changes in the cleaned region. The three-dimensional morphology changes and roughness of the cleaning area were studied using laser scanning confocal microscopy (LSCM). The value of linear roughness was the average of the measurements obtained from five different straight lines, while the value of surface roughness was the average of the measurements from five different points on the plane. The hardness changes in the cleaning area were investigated using the Vickers microhardness tester. The hardness value of each sample was the average of the five test points on the diagonal of the sample. The cleaning mechanism under different laser energy densities was analyzed using high-speed video.

## 2. Materials and Methods

**Figure 1:**
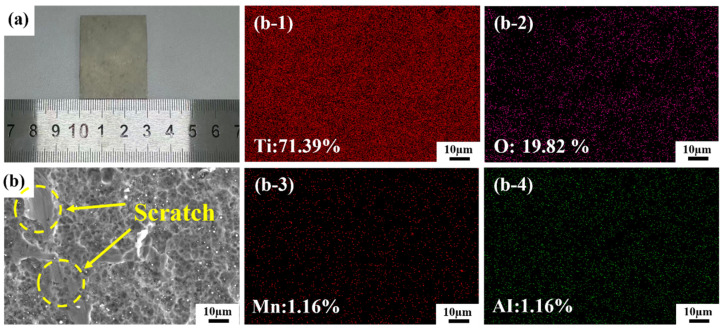
(**a**) Macroscopic morphology of the original sample; (**b-1**) The EDS (Energy Dispersive X-ray Spectroscopy) results of titanium element; sub-figure (**b**) shows the microscopic surface morphology of the original sample. (**b-2**) The EDS results of the oxygen element; (**b-3**) The EDS results of manganese element; (**b-4**) The EDS results of the aluminum element.

The experimental material was TC1 titanium alloy, cut into specimens measuring 30 mm × 40 mm × 1.5 mm. To remove scale, oil residues, and debris generated during hot working and machining processes, the titanium alloy plates underwent acid pickling treatment at the factory, forming a uniform passivation film on their surfaces. The SEM image and EDS results of the original sample are shown in Figure 1. Scratches may form on the sample surface during storage and transportation. EDS results indicate that the oxide layer on the surface of the TC1 titanium alloy contains 71.39 wt% Ti, 19.82 wt% O, 1.16 wt% Mn, and 1.16 wt% Al.

**Figure 2:**
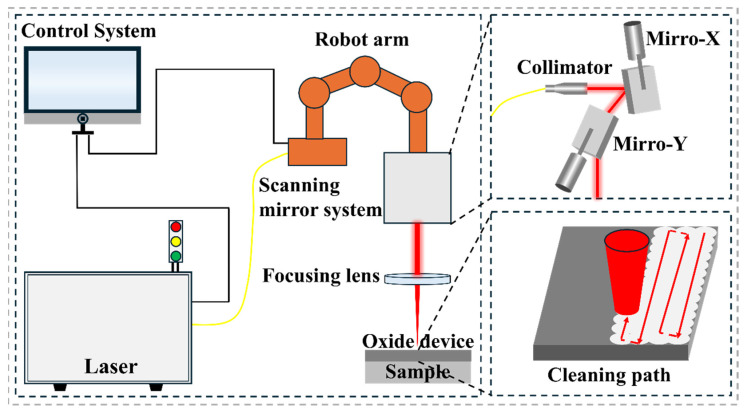
Schematic Diagram of Laser Cleaning Equipment.

The schematic diagram of the laser cleaning equipment is shown in Figure 2. The laser cleaning equipment used in this experiment primarily consists of a control system, fiber laser, laser working head, and robot. A nanosecond pulsed laser (IPG, Marlborough, MA, USA) is employed for direct laser cleaning under atmospheric conditions. The parameters of the laser cleaning equipment are listed in Table 1.

Numerous parameters influence laser cleaning effectiveness, such as power, repetition rate, galvanometer speed, and laser head movement speed. These process parameters interact with one another. To reduce the volume of process experiments, single-pulse energy density (*F*), *x*-axis spot overlap ratio (*Ux*), and *y*-axis overlap ratio (*Uy*) were introduced for laser cleaning experimental research.

(1) $$F=\frac{P}{f\cdot A}$$

(2) $$Ux=\frac{Lx}{D}=\left(1-\frac{Vs}{D\cdot f}\right)\times 100\%$$

(3) $$Uy=\frac{Ly}{D}\times 100\%$$

In this experiment, the overlap ratio of the *x*-axis spot was set equal to the *y*-axis overlap ratio, i.e., *Ux* = *Uy*. The experimental parameters are shown in Table 2.

Surface morphology and chemical elements of samples before and after cleaning were analyzed using a field emission scanning electron microscope (FE-SEM, Zeiss, Melin Compact, Auberkheim, Germany) coupled with an energy-dispersive spectrometer (EDS, Oxford Instruments, X-MaxN, Oxford, UK). X-ray photoelectron spectroscopy (XPS, ESCALAB 250Xi, Thermo Fisher, MA, USA) was employed to analyze the valence state changes of Ti and O on the sample surface before and after cleaning. XPS data were peak-corrected using the C 1s binding energy (284.8 eV), and all data were fitted using Avantage 5.9 software. The surface roughness and three-dimensional topography of the aforementioned samples were measured using an optical laser scanning confocal microscope (OLS-5100, Hitachi Olympus, Tokyo, Japan). Additionally, a high-speed camera capable of capturing images at 4000 frames per second was employed to record the dynamic behavior during the cleaning process.

## 3. Results and Discussion

### 3.1. Macroscopic Morphology Analysis

**Figure 3:**
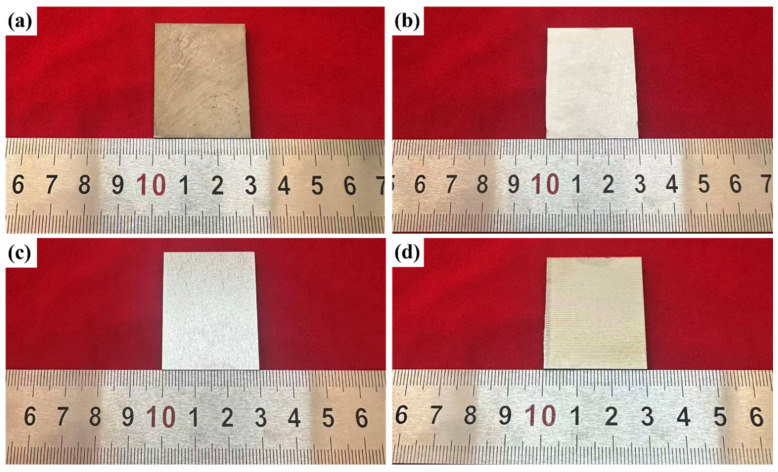
Macroscopic morphology of specimens after cleaning at different laser energy densities: (**a**) original sample; (**b**) 2.55 J/cm^2^; (**c**) 6.37 J/cm^2^; (**d**) 8.91 J/cm^2^.

To investigate the influence of laser energy density on the cleaning efficacy for oxide layers on the surface of TC1 titanium alloy, this study conducted laser cleaning experiments at three distinct energy densities: 2.55 J/cm^2^, 6.37 J/cm^2^, and 8.91 J/cm^2^. The results were compared with the macroscopic morphology of the as-received (uncleaned) specimens, as presented in Figure 3. The as-received specimen surface exhibited a grayish-black appearance, with visible contaminants—including grease, particulate debris, and other residues—introduced during transportation and mechanical cutting. Upon laser cleaning at 2.55 J/cm^2^, the surface attained a uniform silver-gray appearance, and most surface contaminants were effectively removed, confirming that effective cleaning is achievable at this energy density. When the laser energy density was increased to 6.37 J/cm^2^, the surface transformed into a bright silvery-white color with pronounced metallic luster, indicating a substantial improvement in cleaning performance. This condition was preliminarily identified as the optimal cleaning parameter. However, further increasing the laser energy density to 8.91 J/cm^2^ induced excessive ablation, leading to severe secondary oxidation of the titanium alloy surface and resulting in a yellowish-brown discoloration. Subsequent investigations will integrate microstructural characterization and compositional analysis to elucidate the underlying cleaning mechanisms across the tested laser energy densities.

### 3.2. Surface Composition and Microstructure Analysis

The macroscopic morphology after laser cleaning provides an initial assessment of cleaning effectiveness, while precise characterization requires comprehensive analysis combining surface elemental composition and microstructure. The pickling and passivation film on TC1 titanium alloy primarily consists of TiO^2^ and low-valent titanium oxides. Therefore, the oxygen and titanium elemental content on the cleaned specimen surface can serve as indicators for evaluating cleaning performance.

**Figure 4:**
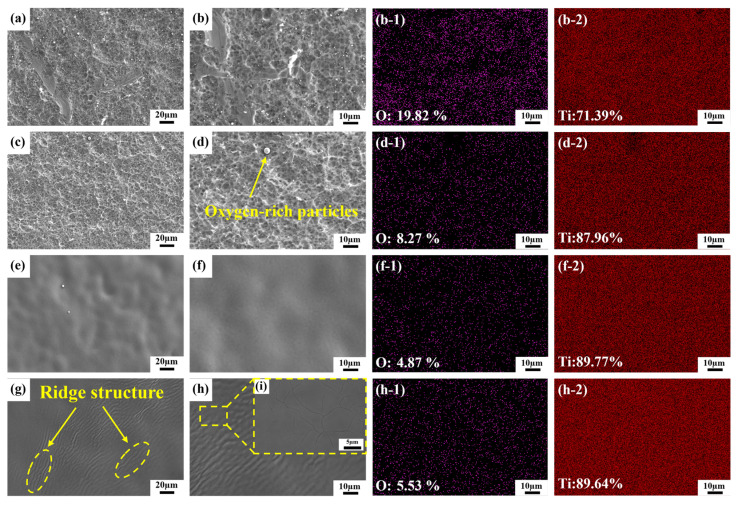
The microscopic surface morphology and EDS results of the samples before and after laser cleaning: (**a**,**b**) original sample, (**b-1**) Oxygen element EDS results of the original sample; (**b-2**) EDS results of titanium element for the original sample; (**c**,**d**) 2.55 J/cm^2^; (**d-1**) Oxygen element EDS results at 2.55 J/cm^2^; (**d-2**) EDS results of titanium element at 2.55 J/cm^2^; (**e**,**f**) 6.37 J/cm^2^; (**f-1**) Oxygen element EDS results at 6.37 J/cm^2^; (**f-2**) EDS results of titanium element at 6.37 J/cm^2^; (**g**–**i**) 8.91 J/cm^2^; (**h-1**) Oxygen element EDS results at 8.91 J/cm^2^; (**h-2**) EDS results of titanium element at 8.91 J/cm^2^.

Figure 4 presents the SEM morphologies and EDS elemental analysis results of the TC1 titanium alloy specimen surface before and after laser cleaning. At a laser energy density of 2.55 J/cm^2^, fine oxygen-rich particles on the specimen surface were effectively removed. EDS analysis revealed a substantial reduction in surface oxygen content. However, the applied laser energy was insufficient to fully ablate the entire oxide layer. Consequently, the molten oxide layer resolidified on the specimen surface, forming a characteristic network-like structure (Figure 4c). Concurrently, a small fraction of the molten oxide was ejected by the laser-induced recoil pressure and subsequently redeposited onto the surface as oxygen-rich spherical particles (Figure 4d). When the laser energy density was increased to 6.37 J/cm^2^, the oxide layer was completely removed. The underlying titanium alloy substrate underwent localized melting and exhibited excellent melt fluidity; upon rapid solidification and recrystallization, it yielded a relatively flat and smooth surface (Figure 4f). At a laser energy density of 8.91 J/cm^2^, although the surface oxide layer was entirely eliminated, the excessively high energy input induced pronounced thermal effects in the TC1 titanium alloy substrate, leading to secondary oxidation during cooling. Upon solidification, this resulted in the formation of ridge-like topographic features (Figure 4g). Moreover, the intense, transient laser irradiation caused rapid heating and subsequent quenching of the substrate within an extremely short time frame, generating a steep temperature gradient. This thermal stress ultimately triggered the formation of surface microcracks (Figure 4i).

**Figure 5:**
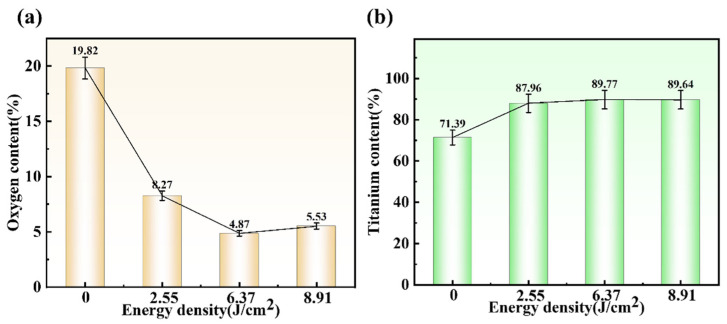
Elemental content on the surface of specimens after cleaning with different laser power densities: (**a**) oxygen; (**b**) titanium.

Figure 5 shows the distribution of oxygen and titanium elements on the surface of the samples after cleaning with different laser energy densities. Here, the content of both oxygen and titanium elements is the average value of the measurements taken at five different points. As shown in the figure, when the laser energy density increased from 0 to 2.55 joules per square centimeter, the oxygen content decreased significantly from 19.82% to 8.27%. This indicates that the oxide layer on the surface of the titanium alloy has been preliminarily removed. When the laser energy density increased to 6.37 J/cm^2^, the oxygen content dropped to a minimum of 4.87%, representing a 14.95% decrease compared to the unwashed sample surface. This indicates that the surface oxide layer was effectively removed. At a laser energy density of 8.91 J/cm^2^, secondary oxidation of the substrate caused the oxygen content to rebound to 5.53%. The titanium content on the unwashed sample surface was 71.39%. As the laser energy density gradually increased, the titanium content exhibited an overall trend of first rising and then stabilizing. When the laser energy density exceeded 6.37 J/cm^2^, the titanium content remained essentially constant.

### 3.3. XPS Analysis

**Figure 6:**
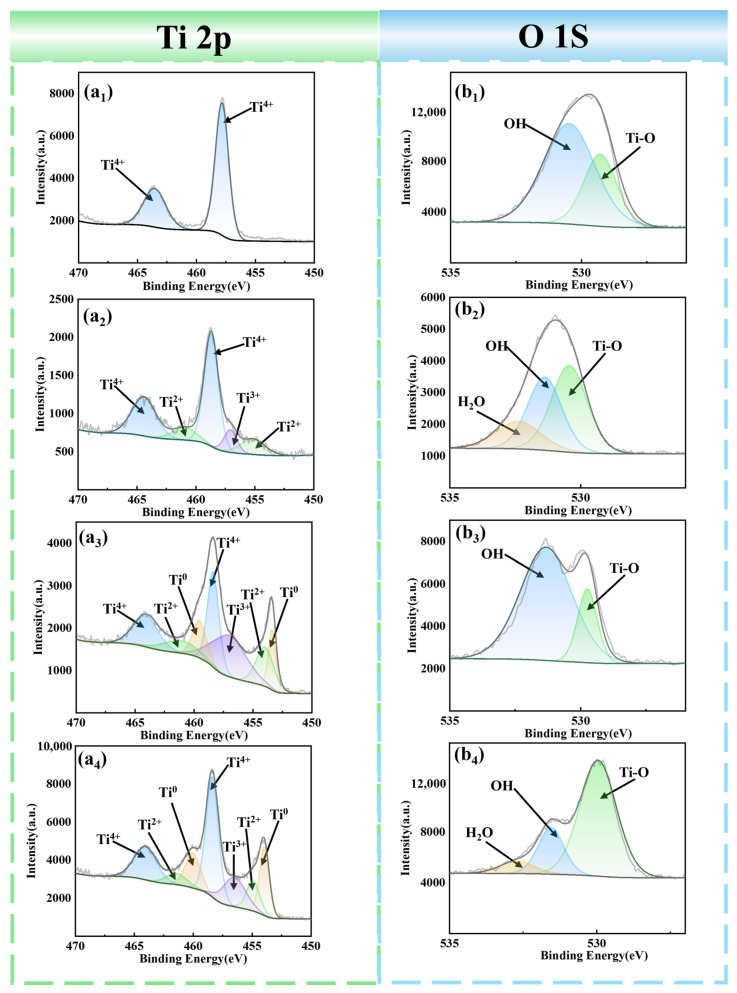
XPS spectra of sample surfaces after cleaning with different laser energy densities: (**a_1_**,**b_1_**) original sample; (**a_2_**,**b_2_**) 2.55 J/cm^2^; (**a_3_**,**b_3_**) 6.37 J/cm^2^; (**a_4_**,**b_4_**) 8.91 J/cm^2^.

To clarify the phase composition of the sample surface before and after cleaning, this study conducted XPS analysis on the original sample and the samples cleaned with three laser energy densities. Figure 6(a_1_–a_4_) show the XPS fitting spectra of Ti 2p for the four samples, revealing four valence states of Ti: Ti^4+^, Ti^3+^, Ti^2+^, and Ti^0^. The 2p_3_/_2_ peak positions are distributed at 458.47 ± 0.24 eV, 457.5 ± 0.2 eV, 456.18 ± 0.06 eV, and 453.92 ± 0.01 eV, while the 2p_1_/_2_ peak positions are at 464.27 ± 0.22 eV, 463.5 ± 0.2 eV, and 460.27 ± 0.08 eV [24,25,26]. Figure 6(b_1_–b_4_) present the XPS fitting spectra of O 1s for the four samples. The O 1s fitting peaks consist of three sub-peaks, corresponding to 530.01 ± 0.19 eV (Ti–O bond), 531.68 ± 0.24 eV (hydroxyl bond), and 533.05 ± 0.03 eV (H_2_O) [27]. Only Ti^4+^ characteristic signals were detected on the surface of the original sample, because the main component of the passive film formed on the TC1 titanium alloy after pickling is TiO_2_. At a laser energy density of 2.55 J/cm^2^, the intensity of the Ti^4+^ characteristic signal decreased; at this point, the relative atomic percentage of Ti^4+^ was 75.73%, while characteristic signals of Ti^2+^ and Ti^3+^ began to appear, but no Ti^0^ signal was observed, indicating that the titanium alloy substrate was not exposed. At a laser energy density of 6.37 J/cm^2^, the Ti^0^ signal appeared, and the intensities of both the Ti^4+^ characteristic signal and the Ti–O characteristic signal decreased significantly. The relative atomic percentage of Ti^4+^ dropped from 75.73% to 35.46%, proving that the surface oxide film had been effectively removed. The appearance of the Ti^0^ characteristic signal indicated that the TC1 titanium alloy substrate was exposed, corresponding to the EDS results in Figure 4. At this energy density, the oxygen content decreased to its lowest level, achieving the best cleaning effect. At a laser energy density of 8.91 J/cm^2^, the intensity of the Ti^4+^ characteristic signal rebounded, and the relative atomic percentage of Ti^4+^ rose back to 47.36%. This is because although an excessively high laser energy density can instantly remove the surface oxide film, a large amount of heat accumulates on the sample surface during irradiation, leading to severe secondary oxidation of the sample and the reformation of TiO_2_ on the surface. The intensity of the Ti–O characteristic signal first decreased and then increased sharply, further demonstrating the secondary oxidation behavior of the sample surface under high laser energy density. Corresponding to the EDS results in Figure 4, at this laser energy density, the oxygen content increased from 4.87% to 5.53%, further confirming the secondary oxidation phenomenon.

### 3.4. Three-Dimensional Morphology and Surface Roughness Analysis

**Figure 7:**
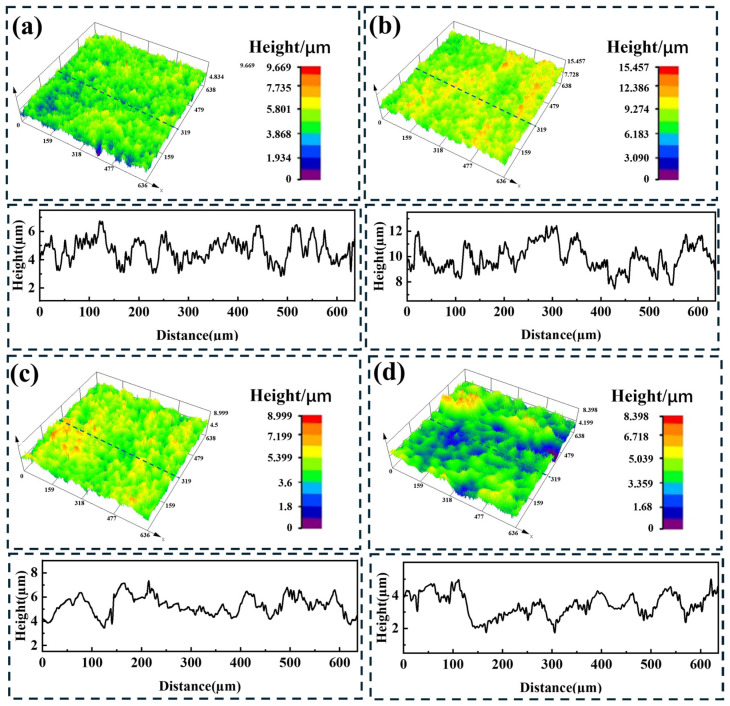
Three-dimensional topography and line roughness distribution of specimen surfaces after cleaning at different laser energy densities: (**a**) original sample; (**b**) 2.55 J/cm^2^; (**c**) 6.37 J/cm^2^; (**d**) 8.91 J/cm^2^.

**Figure 8:**
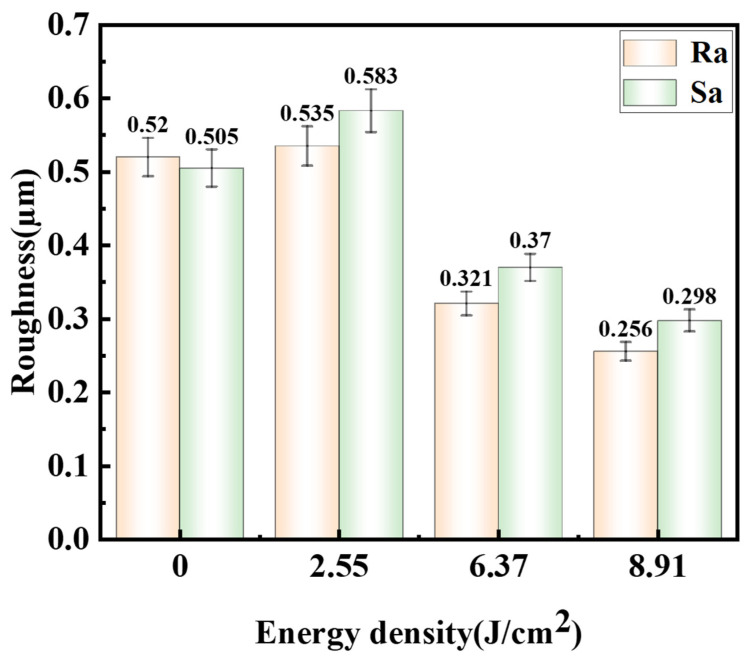
Surface roughness of specimens after cleaning with different laser energy densities.

The surface roughness of the sample after laser cleaning has a significant effect on welding quality [28]. To obtain high-quality welded joints, it is necessary to study the three-dimensional morphology and roughness of the sample surface after cleaning. Figure 7 shows the three-dimensional surface morphologies of the samples after laser cleaning at different laser energy densities, obtained using a laser confocal microscope. Figure 8 presents the surface roughness of the samples after cleaning at different laser energy densities, where Ra represents the line roughness and Sa represents the area roughness. During transportation, the titanium alloy sample surface exhibits scratches, impacts, pits, and defects, with an area roughness of 0.505 µm and a corresponding line roughness of 0.52 µm. As the laser energy density gradually increases, the surface roughness first increases and then decreases. When the surface temperature is slightly higher than the melting point but lower than the boiling point of the oxide film, some of the oxide film will peel off, while the remaining undeployed oxide film will re-solidify to form protrusions, thereby creating a network structure on the surface of the sample. This structure exhibits height differences, leading to an increase in surface roughness. At this stage, the roughness reaches its maximum, with an area roughness of 0.597 µm and a corresponding line roughness of 0.534 µm. At a laser energy density of 6.37 J/cm^2^, the surface temperature exceeds the boiling point of the oxide film, causing rapid vaporization of the surface oxide film and generating plasma. The high-temperature, high-pressure metal vapor plasma exerts a recoil pressure on the sample surface, promoting the flow and spreading of molten material, thereby reducing the roughness. The area roughness decreases to 0.370 µm, the profile line scan curve becomes relatively smooth, and the number of peaks and valleys decreases, with a corresponding line roughness of 0.321 µm. At a laser energy density of 8.91 J/cm^2^, the laser energy further increases, and the recoil pressure exerted by the metal vapor plasma on the sample surface becomes larger, resulting in a smoother sample surface. The area roughness and line roughness decrease to 0.370 µm and 0.256 µm, respectively. Although a periodic regular ridge-like structure appears on the sample surface, the height difference within the structure is small, so it does not cause an increase in roughness.

### 3.5. Surface Hardness Analysis

**Figure 9:**
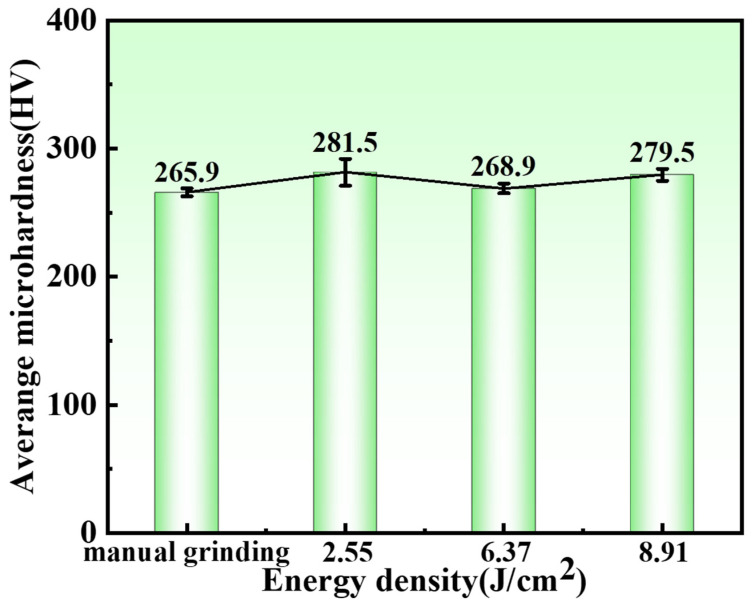
Average Surface Hardness of Specimens at Different Laser Energy Densities.

When the laser acts on the sample surface, it not only removes the oxide film on the titanium alloy surface but also affects the titanium alloy substrate to a certain extent, altering the surface hardness of the sample. Figure 9 shows the trend of the average surface hardness of the samples under different laser energy densities. The average surface hardness of the sample after manual polishing is 265.9 HV. At a laser energy density of 2.55 J/cm^2^, the average surface hardness of the sample increases slightly to 281.5 HV, indicating that the laser treatment slightly improves the surface hardness. This can be attributed to grain refinement and dislocation generation in the remelted layer on the sample surface induced by the laser action, which slightly increases the hardness. Liu et al. [29] used a high-power Nd:YAG laser to remove the corrosion layer on the surface of 304L stainless steel and studied the effect of laser cleaning on the mechanical properties of 304L stainless steel. The study found that after cleaning, the dislocation density increased, accompanied by the introduction of a dense dislocation structure, deformation twins, grain refinement, and precipitation, which changed the mechanical properties of the stainless steel in terms of hardness and tensile strength. As the laser energy further increases to the optimal energy density of 6.37 J/cm^2^, the surface hardness of the sample decreases but remains slightly higher than that after manual polishing, with a hardness value of 268.9 HV. At a laser energy density of 8.91 J/cm^2^, the average surface hardness of the sample is 279.5 HV. Wang et al. [30] found that when cleaning the oxide film on the surface of 5083 aluminum alloy, a high spot overlap ratio causes the aluminum matrix to react with air to form a new oxide film, increasing the microhardness of the aluminum alloy surface to 86.8 HV. At this stage, the newly formed oxide film on the aluminum alloy surface plays a dominant role in improving the surface microhardness. This explains why the sample surface hardness increases at a laser energy density of 8.91 J/cm^2^: the high laser energy density causes severe secondary oxidation, and the newly formed oxide film has a higher hardness, thereby increasing the surface hardness of the sample.

### 3.6. Dynamic Removal Behavior Analysis

**Figure 10:**
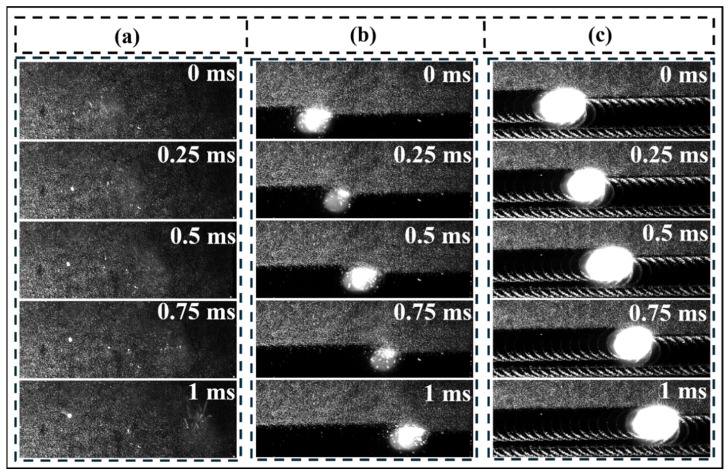
Dynamic behavior of laser cleaning: (**a**) 2.55 J/cm^2^; (**b**) 6.37 J/cm^2^; (**c**) 8.91 J/cm^2^.

To reveal the influence of different laser fluences on the dynamic behavior during the cleaning process, Figure 10 illustrates the dynamic behavior during laser cleaning. In Figure 10b,c, the bright spots represent the plasma plume generated by the interaction between the laser and the oxide film. The area above the demarcation line is the uncleaned region, while the area below is the cleaned region. When the laser fluence is low (Figure 10a), the surface temperature of the sample is slightly above the melting point of the oxide film but below its boiling point, allowing only partial removal of the oxide film. The molten oxide film is ejected as granular spatter under laser action, and the sample surface changes from dark gray to light gray. As the laser fluence gradually increases (Figure 10b,c), the surface temperature of the sample exceeds the boiling point of the oxide layer. Laser irradiation promotes the decomposition and vaporization of the oxide film and surface contaminants, generating plasma. As shown in Figure 10b, when the laser fluence is 6.37 J/cm^2^, the cleaned area is flat and smooth, exhibiting a metallic black luster. When the laser fluence increases to 8.91 J/cm^2^, under the combined effect of excessive energy and surface tension, the molten metal on the surface is displaced outward. After rapid cooling, the molten metal forms a periodic ridge structure, consistent with the morphology observed on the macroscopic sample surface in Figure 4f. The dynamic behavior of laser cleaning further demonstrates that, under appropriate laser fluence, the oxide film can be removed while minimizing damage to the surface morphology. Furthermore, the differences in dynamic behavior during cleaning under different fluences also imply variations in the cleaning mechanisms.

**Figure 11:**
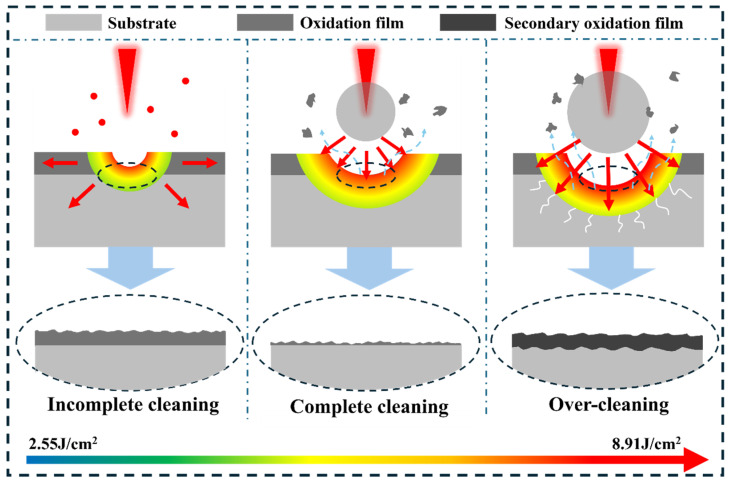
Laser Removal Mechanism of the oxide layer on TC1 Titanium Alloy.

Laser removal of oxide layers from titanium alloy surfaces is a complex physicochemical coupling process involving the synergistic interaction of multiple physical fields, including light, heat, and mechanical forces. Laser cleaning involves multiple mechanisms, primarily including laser ablation, thermal expansion, stress vibration, phase explosion, and plasma impact, based on the interaction characteristics between the laser and contaminants or substrates. The corresponding cleaning mechanisms also vary under different laser energy densities. Figure 11 illustrates the laser cleaning mechanisms at different laser energy densities.

Figure 11 illustrates the oxide layer removal mechanism at different laser energy densities. In this study, when the laser energy density reached 2.55 J/cm^2^, the sample surface temperature slightly exceeded the oxide layer ‘s melting point but did not reach its boiling point. At this stage, the cleaning mechanism primarily involved laser ablation. After partial oxide layer detachment, the residual film underwent melting-solidification behavior, forming a network structure on the sample surface. Simultaneously, a small amount of molten oxide layer was splattered and deposited under laser irradiation, forming oxygen-rich particles. When the laser energy density reaches 6.37 J/cm^2^, the surface temperature of the specimen exceeds the boiling point of the oxide layer. High-energy laser irradiation causes the instantaneous decomposition and vaporization of the oxide layer and surface contaminants, forming plasma. The appearance of the plasma in Figure 10b signifies a shift in the cleaning mechanism from laser ablation to plasma impact. The plasma exerts pressure on the melt pool at the specimen surface, promoting its movement and facilitating surface topography flattening (Figure 4f). The oxygen content at the specimen surface reached its minimum value of 4.87% during this process (Figure 5). Wang et al. [31] also observed favorable results when cleaning the TC4 titanium alloy oxide layer in air. This occurs because laser irradiation causes evaporation and vaporization of the oxide layer, ionizing the overlying metallic gas to form plasma. The sudden high-temperature, high-pressure plasma temporarily isolates the molten substrate from external air, mitigating secondary oxidation to some extent. When the laser energy density increases to 8.91 J/cm^2^, the plasma volume expands with rising laser energy density. Excessive thermal input causes severe secondary oxidation of the titanium alloy substrate, accompanied by crack initiation on the surface [32]. Therefore, selecting an appropriate laser energy density is crucial for effectively removing the oxide layer from titanium alloys.

## 4. Conclusions

This study investigated the removal of oxide layers from the surface of TC1 titanium alloy using nanosecond pulsed lasers. It clarified the cleaning effectiveness under different laser energy densities and revealed the impact of varying laser energy densities on mechanical properties post-cleaning, along with the mechanism of oxide layer removal. The main conclusions are as follows:

(1) At a pressure of 6.37 J/cm^2^, the oxide film of TC1 titanium alloy was successfully removed while maintaining the integrity of the substrate. The XPS results showed that the intensity of the Ti^0^ peak was the highest, while the intensity of the Ti^4+^ peak decreased significantly, and the oxygen content dropped to the lowest level of 4.87%. The sample presented a metallic lustrous silver-white color.
(2) At 6.37 J/cm^2^, surface quality was optimal. While the oxide layer was removed, the molten titanium alloy matrix exhibited excellent fluidity. After recrystallization, a relatively flat and smooth surface was obtained, with the specimen’s surface roughness significantly reduced to 0.37 µm.
(3) After manual polishing, the surface hardness value of the specimen was 265.9 HV. Following cleaning at the optimal energy density, the surface hardness value increased to 268.9 HV, indicating that laser cleaning slightly enhanced the surface hardness of the titanium alloy. Laser cleaning had minimal adverse effects on the mechanical properties of the titanium alloy substrate and can serve as an alternative method for oxide layer removal, replacing traditional manual polishing.
(4) The removal mechanism of the oxide layer on TC1 titanium alloy primarily involves laser ablation and plasma impact. At lower laser energy densities (2.55 J/cm^2^–6.37 J/cm^2^), laser ablation dominates the removal process. At higher laser energy densities (6.37 J/cm^2^–8.91 J/cm^2^), plasma impact becomes the predominant mechanism, leading to secondary oxidation phenomena.

## Figures and Tables

**Table 1:** Laser Cleaning Equipment Parameters.

Name	Parameter Value
Wavelength (nm)	1064
Power (W)	10–1000
Pulse width (ns)	100
Repetition frequency (kHz)	10
Diameter of the focused spot (mm)	1
Mirror rotation speed (mm/s)	100–10,000
Step distance (µm)	5–10,000
Scanning width (mm)	10–100
Laser head movement speed (mm/s)	0–10,000
Scanning format (mm^2^)	100 × 100

**Table 2:** Experimental Parameters for Laser Cleaning.

Parameters	Parameter Value
Single-pulse energy density (J/cm^2^)	2.55, 6.37, 8.91
The overlap rate of light spots on the *x*/*y* axes (%)	70
Frequency (kHz)	10

## Data Availability

The data presented in this study are available on request from the corresponding author.
